# GLP-1-Based Pharmacotherapies and Skeletal Muscle Health in Obesity: A Narrative Review of Body Composition, Muscle Function, and Sarcopenia-Related Outcomes

**DOI:** 10.3390/nu18193247

**Published:** 2026-10-01

**Authors:** Justyna Knapik, Weronika Fic, Ewelina Polak-Szczybyło

**Affiliations:** 1 Student Scientific Club of Human Nutrition, Faculty of Health Sciences and Psychology, Collegium Medicum, University of Rzeszów, ul. Warzywna 1a, 35-959 Rzeszow, Poland; jk122124@stud.ur.edu.pl (J.K.);wf130811@stud.ur.edu.pl (W.F.); 2 Departament of Dietetics, Faculty of Health Sciences and Psychology, Collegium Medicum, University of Rzeszów, ul. Warzywna 1a, 35-959 Rzeszow, Poland

**Keywords:** obesity, glucagon-like peptide-1 receptor agonists, GLP-1RAs, sarcopenia, skeletal muscle, fat-free mass, muscle strength, body composition

## Abstract

**Background:** Obesity is associated with impaired skeletal muscle health and sarcopenic obesity. GLP-1-based pharmacotherapies induce weight loss, but accompanying reductions in fat-free or lean mass raise concerns about skeletal muscle loss. Conversely, reductions in adiposity, inflammation, insulin resistance, and intramuscular fat may be beneficial. Objective: This narrative review evaluated the effects of GLP-1-based pharmacotherapies on body composition, muscle quantity, composition and function, and their potential role in sarcopenia. **Methods:** PubMed and Google Scholar were searched for clinical and experimental studies published primarily between 2016 and 2026. **Results:** Preclinical studies identified potentially protective mechanisms, including improved insulin sensitivity, reduced inflammation, and modulation of mitochondrial function and protein turnover, but some also indicated muscle loss or proteolysis. Clinical studies reported greater reductions in fat mass than in fat-free or lean mass, neither of which represents skeletal muscle exclusively. Direct muscle and functional outcomes were assessed in few studies and yielded mixed results. Incident sarcopenia was rarely evaluated. **Conclusions:** GLP-1-based pharmacotherapies may influence sarcopenia risk through competing pathways. Metabolic improvements may support muscle health, whereas energy restriction, inadequate protein intake, gastrointestinal adverse effects, and lean tissue loss may increase vulnerability. Current evidence is insufficient to determine their net effect on sarcopenia risk.

## 1. Introduction

Obesity is one of the greatest public health challenges of the twenty-first century. It is a complex, multifactorial chronic disease characterized by excessive adipose tissue accumulation resulting from a sustained positive energy balance [1]. Its development is driven by intricate interactions among genetic, biological, environmental, and behavioral factors. Obesity is associated with an increased risk of numerous chronic diseases, including type 2 diabetes mellitus, cardiovascular disease, several types of cancer, and premature mortality [2]. Recent advances in obesity pharmacotherapy have substantially improved treatment outcomes. Among currently available anti-obesity medications, glucagon-like peptide-1 receptor agonists (GLP-1RAs) have demonstrated remarkable efficacy in promoting weight loss while improving glycemic control and other cardiometabolic parameters [3]. However, the reduction in body weight achieved with GLP-1RA therapy is accompanied not only by a decrease in fat mass (FM) but also by a reduction in fat-free mass (FFM), raising concerns regarding its potential impact on skeletal muscle health [4]. Sarcopenia is characterized by progressive loss of skeletal muscle mass, strength, and physical performance, resulting in impaired functional capacity and adverse health outcomes [5,6]. Although reductions in FFM during GLP-1RA therapy have prompted concerns about an increased risk of sarcopenia, growing evidence suggests that changes in muscle quantity may not necessarily reflect deterioration in muscle function or quality. Furthermore, experimental studies indicate that GLP-1RAs may exert direct protective effects on skeletal muscle through multiple molecular pathways, including improved mitochondrial function, attenuation of inflammation, and inhibition of muscle protein degradation. Nevertheless, the available evidence remains inconsistent, and the clinical significance of muscle mass loss during GLP-1RA therapy has not yet been fully elucidated [5].

This narrative review aimed to evaluate the effects of GLP-1–based pharmacotherapies, including GLP-1 receptor agonists and the dual GIP/GLP-1 receptor agonist tirzepatide, on body composition, skeletal muscle mass and quality, muscle strength, physical performance, and other sarcopenia-related outcomes in adults with overweight or obesity.

## 2. Materials and Methods

Emphasis was placed on extracting evidence from preclinical and clinical studies and identifying current knowledge gaps regarding the effects of GLP-1RAs on skeletal muscle health and the potential development of sarcopenia. This narrative review was conducted to synthesize current evidence on the effects of GLP-1-based pharmacotherapies on body composition, skeletal muscle health, and sarcopenia-related outcomes in individuals with overweight or obesity. In this review, the term “GLP-1-based pharmacotherapies” encompasses selective GLP-1 receptor agonists (GLP-1RAs) and the dual GIP/GLP-1 receptor agonist tirzepatide.

Relevant publications were identified collaboratively by the three authors through iterative searches of PubMed/MEDLINE and Google Scholar. PubMed/MEDLINE served as the principal bibliographic database, whereas Google Scholar was used as a supplementary tool for targeted searches, citation tracking, and the identification of additional potentially relevant publications. Reference lists of eligible primary studies and relevant reviews were also screened. Because Google Scholar uses dynamic relevance-based ranking, it was not treated as an independent systematic evidence source, and no claim of exhaustive coverage was made. Searches covered publications from January 2016 to August 2026 and combined terms related to the intervention and outcomes, including “GLP-1 receptor agonist”, “GLP-1RA”, “GLP-1-based therapy”, “semaglutide”, “liraglutide”, “exenatide”, “dulaglutide”, “lixisenatide”, “tirzepatide”, “obesity”, “body composition”, “fat-free mass”, “lean mass”, “appendicular lean mass”, “skeletal muscle”, “muscle mass”, “muscle volume”, “muscle composition”, “intramuscular fat”, “muscle strength”, “physical performance”, “sarcopenia”, and “sarcopenic obesity”. The final search was completed on August 30, 2026. Publications preceding 2016 were used only when required to support established definitions, diagnostic criteria, or foundational biological mechanisms and were not included in the primary evidence synthesis.

Clinical studies were eligible if they: (1) included adults with overweight or obesity; (2) evaluated a GLP-1RA or tirzepatide; and (3) reported at least one relevant outcome, including fat-free mass (FFM), lean mass, appendicular lean mass, skeletal muscle mass, muscle area or volume, intramuscular fat, muscle strength, physical performance, sarcopenia, or sarcopenic obesity. Randomized controlled trials, secondary analyses or substudies of randomized trials, and prospective or retrospective observational studies were eligible. Preclinical studies were included if they evaluated a GLP-1RA or tirzepatide in an animal model or skeletal muscle cell model and reported structural, functional, metabolic, mitochondrial, or molecular muscle-related outcomes. Studies without a relevant muscle or body-composition outcome, conference abstracts without a full-text publication, editorials, commentaries, and publications lacking sufficient information for interpretation were excluded. Reviews and meta-analyses were used to provide context and identify additional primary studies but were not counted as part of the primary clinical or preclinical evidence base.

Eligibility decisions were discussed among the authors and resolved by consensus. Particular attention was given to distinguishing indirect body-composition measures, such as fat-free and lean mass, from direct assessments of skeletal muscle quantity or composition, functional outcomes, and formally defined sarcopenia. No formal study-level risk-of-bias tool was applied. Instead, study design, sample size, comparator, treatment duration, outcome assessment methods, and the directness of the evidence for sarcopenia were considered descriptively during the narrative synthesis. Evidence from controlled clinical studies assessing skeletal muscle quantity, strength, or physical performance was regarded as more directly relevant to muscle health than evidence from uncontrolled studies or studies limited to fat-free or lean mass. Outcomes based solely on fat-free or lean mass were treated as indirect indicators of muscle health, whereas animal and cell studies were used to inform the mechanistic interpretation. A total of 20 original research publications were included in the narrative synthesis: eight preclinical and 12 clinical publications. The clinical evidence comprised primary studies, secondary or prespecified analyses, and body-composition substudies. The clinical evidence included primary trials, observational studies, and secondary analyses or body-composition substudies; therefore, the number of publications does not correspond to the number of fully independent study populations. Owing to substantial heterogeneity in study design, populations, interventions, assessment methods, and outcomes, the findings were synthesized narratively.

As the literature identification process was iterative and conducted for a narrative rather than a systematic review, record-level counts at the initial retrieval and title-screening stages were not prospectively documented.

## 3. Obesity

Obesity is a chronic disease affecting children, adolescents, and adults and currently represents one of the greatest public health challenges worldwide [7]. According to the World Health Organization (WHO), approximately 890 million adults were living with obesity in 2022, and its global prevalence has more than doubled since 1990 [2]. In its simplest form, the increasing prevalence of obesity is driven by a sustained positive energy balance resulting from excessive energy intake relative to energy expenditure, often accompanied by insufficient physical activity [7]. Excess energy is stored as triglycerides within adipose tissue, leading to progressive fat accumulation [8]. Obesity is a complex, multifactorial disease that may develop from early childhood and exert long-term adverse effects on health throughout the lifespan [1]. Its development results from intricate interactions between environmental and genetic factors. These include dietary habits, physical activity, sex, ethnicity, socioeconomic status, as well as neurological mechanisms involved in appetite regulation and various metabolic and endocrine disturbances [8,9]. Obesity is associated with numerous systemic metabolic abnormalities, including dyslipidemia, oxidative stress, and insulin resistance [8]. Moreover, increasing obesity severity is accompanied by a higher prevalence of metabolic dysfunction-associated steatotic liver disease (MASLD) [10]. Obesity is also closely associated with chronic low-grade inflammation affecting multiple organs and tissues, including adipose tissue, skeletal muscle, pancreatic islets, the liver, brain, and kidneys [7]. If left untreated, obesity substantially increases the risk of type 2 diabetes mellitus, cardiovascular disease, and premature mortality [8]. The primary objective of obesity management extends beyond achieving weight loss or a predefined body mass index (BMI). Instead, treatment aims to improve overall health, reduce obesity-related complications, and enhance long-term clinical outcomes. In patients with obesity-related comorbidities, therapeutic interventions seek to improve disease control and, in some cases, achieve remission. Achieving and maintaining clinically meaningful weight loss has been associated with improved quality of life, alleviation of obesity-related symptoms, and reduced risks of cardiovascular events and certain malignancies, ultimately contributing to increased healthy life expectancy [10]. Consequently, a better understanding of the pathophysiological mechanisms underlying obesity is essential for developing more effective therapeutic strategies and preventing obesity-related complications [7]. Lifestyle modification remains the cornerstone of obesity treatment, with nutritional therapy serving as a fundamental component that can be combined with other therapeutic approaches. The greatest clinical benefits are achieved through a comprehensive, multidisciplinary strategy integrating dietary intervention, regular physical activity, behavioral modification, psychological support, pharmacotherapy, and bariatric surgery when clinically indicated [10]. Nutritional management aims to establish a sustainable energy deficit while ensuring adequate intake of essential nutrients. Therefore, excessively restrictive calorie-restricted diets or nutritionally inadequate dietary patterns are not recommended. Instead, dietary recommendations should be individualized according to the patient’s health status, nutritional requirements, and personal preferences to maximize long-term adherence. Regular physical activity represents both an integral component of obesity treatment and a cornerstone of a healthy lifestyle. Its benefits extend beyond weight reduction and include improvements in cardiorespiratory fitness, glycemic control, blood pressure regulation, and overall well-being. Individuals with a sedentary lifestyle are encouraged to gradually increase their level of physical activity by incorporating both aerobic and resistance exercise, with the long-term goal of establishing sustainable exercise habits [10]. Psychological and behavioral factors also play a crucial role in comprehensive obesity management. Individuals living with obesity frequently experience body image dissatisfaction and weight stigma, both of which may adversely affect mental health. Evidence suggests that obesity-related stereotypes and stigmatizing attitudes do not facilitate successful weight management; conversely, they may contribute to further weight gain and poorer psychological well-being [11]. Psychological interventions can assist patients in identifying emotional and behavioral factors influencing eating habits, lifestyle behaviors, and treatment adherence. Furthermore, psychological status has been shown to influence the outcomes of bariatric surgery, underscoring the importance of integrating psychological support into comprehensive obesity care [12]. Pharmacotherapy is recommended for patients who meet established clinical criteria [10]. In recent years, GLP-1RAs have emerged as one of the most effective pharmacological treatments for obesity [3]. Although these agents produce substantial and sustained weight loss, increasing attention has been directed toward their effects on FFM and the potential implications for skeletal muscle health and the development of sarcopenia [4]. For patients with severe obesity, bariatric surgery remains the most effective intervention for achieving long-term weight loss, with its efficacy well documented in numerous clinical studies. However, bariatric treatment extends beyond the surgical procedure itself and requires comprehensive perioperative care, including nutritional education, psychological support, and structured physical rehabilitation. Rather than representing an alternative to pharmacotherapy, bariatric surgery and anti-obesity medications should be regarded as complementary treatment modalities that may be integrated to optimize long-term weight management and improve overall clinical outcomes.

## 4. Sarcopenia

Sarcopenia is a progressive skeletal muscle disorder characterized by the loss of muscle mass, strength, and physical function [6]. The term sarcopenia was first introduced by Irwin Rosenberg to describe the age-related decline in skeletal muscle mass [13]. Before the publication of the European Working Group on Sarcopenia in Older People (EWGSOP) consensus in 2010, the diagnosis of sarcopenia was based primarily on the assessment of reduced muscle mass. However, this approach was considered insufficient because it failed to account for muscle function. Although several definitions of sarcopenia have since been proposed, the revised EWGSOP2 consensus remains the most widely accepted diagnostic framework [6,14]. According to the EWGSOP2 definition, sarcopenia is a progressive and generalized skeletal muscle disorder associated with an increased risk of adverse outcomes, including falls, fractures, physical disability, and mortality. The updated consensus emphasizes that muscle strength is a better predictor of adverse clinical outcomes than muscle mass alone. Consequently, low muscle strength has been established as the primary criterion for identifying probable sarcopenia and is considered the most reliable indicator of muscle function. The diagnosis of sarcopenia is confirmed by the presence of reduced muscle quantity or quality in addition to low muscle strength, whereas the coexistence of impaired physical performance indicates severe sarcopenia [14]. The aging process disrupts skeletal muscle homeostasis, leading to an imbalance between anabolic and catabolic processes that favors progressive muscle loss. Sarcopenia is characterized by a reduction in the number and cross-sectional area of type II muscle fibers, increased intramuscular fat infiltration, and impaired regenerative capacity resulting from a decline in both the number and activity of satellite cells. The pathogenesis of sarcopenia is multifactorial and extends beyond chronological aging. Chronic low-grade inflammation, insulin resistance, mitochondrial dysfunction, and impaired neuromuscular junction function have all been identified as key contributors to disease development [15]. Sarcopenia predominantly affects older adults, although it may also occur in middle-aged individuals and in association with chronic diseases [6]. Its prevalence among community-dwelling older adults has been estimated to reach approximately 29%, whereas among individuals aged 80 years or older it ranges from 11% to 50%, depending on the diagnostic criteria and study population [15]. Risk factors include low levels of physical activity, prolonged immobilization, and chronic diseases such as type 2 diabetes mellitus, chronic kidney disease, chronic liver disease, metabolic disorders, and cancer [6,14,16]. Nutritional factors also play a crucial role, as inadequate energy and protein intake resulting from anorexia, malabsorption, limited access to nutritionally adequate food, or impaired food intake may accelerate the decline in skeletal muscle mass and function [14]. More recently, reductions in lean body mass during GLP-1RA treatment have raised concerns regarding potential adverse effects on skeletal muscle health. However, lean body mass is not equivalent to skeletal muscle mass, and its reduction alone cannot establish the development of sarcopenia [5]. A recent meta-analysis by Yuan and Larsson demonstrated that sarcopenia is associated with an increased risk of several adverse health conditions, including osteoporosis, cognitive impairment, metabolic syndrome, hypertension, diabetes mellitus, depression, falls, and fractures. However, the causal nature of these associations remains uncertain because of potential confounding factors, methodological limitations, differences in diagnostic criteria, and substantial heterogeneity among the included studies. Among the reported associations, the relationship between sarcopenia and an increased risk of falls appears to be the most consistent and robust, regardless of the diagnostic definition applied [6]. The terms fat-free mass, lean mass, skeletal muscle mass, muscle volume, and muscle quality describe distinct constructs and should not be used interchangeably. FFM comprises all non-fat components of the body, including skeletal muscle, organs, bone, and body water, whereas lean mass is dependent on the terminology and methodology used by the measurement device. Skeletal muscle mass refers more specifically to muscle tissue, while muscle volume represents a regional anatomical measurement usually obtained using MRI or CT. Muscle quality describes characteristics such as intramuscular fat infiltration, muscle density, or force relative to muscle quantity rather than muscle quantity itself. Accordingly, changes in fat-free or lean mass were interpreted as indirect indicators, changes in skeletal muscle mass or volume as measures of muscle quantity, and muscle quality, strength, and physical performance as separate outcomes [14,17,18,19].

## 5. Sarcopenic Obesity

The European Society for Clinical Nutrition and Metabolism (ESPEN) and the European Association for the Study of Obesity (EASO) define sarcopenic obesity (SO) as the coexistence of obesity and sarcopenia [20]. Importantly, SO should be regarded as a distinct clinical condition rather than merely the coincidental coexistence of these two disorders [21]. Sarcopenic obesity may affect older adults with obesity, individuals with chronic diseases or disabilities, and those who have experienced rapid weight loss [22]. Studies applying the ESPEN/EASO definition indicate that the prevalence of SO ranges from 7.9% to 23% in clinical settings and from 7.1% to 9.6% in non-clinical settings [23]. The pathophysiology of SO is multifactorial and remains incompletely understood. Its underlying mechanisms involve metabolic and hormonal disturbances arising from both ageing and adipose tissue hypertrophy [21,22]. Ageing is associated with declining levels of key anabolic hormones, including testosterone, oestrogen, growth hormone (GH), insulin-like growth factor 1 (IGF-1), and thyroid hormones, accompanied by increased glucocorticoid activity, activation of the renin–angiotensin–aldosterone system (RAAS), and enhanced sympathoadrenal signalling. These changes promote insulin resistance, muscle catabolism, and fat accumulation. In turn, hypertrophic adipocytes release free fatty acids, contributing to their deposition in skeletal muscle as intramyocellular lipids [21,22] and intermuscular adipose tissue, as well as to the accumulation of lipid derivatives such as ceramides and diacylglycerols (DAGs). These processes further exacerbate insulin resistance and contribute to the reduction in muscle mass observed in obesity [21]. At the same time, adipose tissue secretes pro-inflammatory cytokines, which are associated with reduced myokine secretion [21,22]. The accompanying inflammatory state also affects mitochondrial fatty acid oxidation, contributing to increased production of reactive oxygen species (ROS) [21] and mitochondrial dysfunction [21,22]. These mechanisms reinforce one another, creating a vicious cycle that drives the progression of SO [20,21]. The consequences of SO may include metabolic disorders, disability associated with high FM and/or low muscle mass, cardiovascular disease, and respiratory disorders. To reduce the risk of adverse health outcomes, SO should be identified at an early stage. ESPEN and EASO propose that the diagnostic process should begin with screening based on the assessment of BMI or waist circumference using ethnicity-specific cut-off values, together with surrogate indicators of sarcopenia. These indicators include age above 70 years, a diagnosis of chronic disease or cancer, recent hospitalization, recent prolonged immobilization or reduced mobility, a recent history of reduced food intake, recent body weight fluctuations, previous bariatric surgery, and fatigue. Following a positive screening result, a diagnostic assessment should be performed, including the evaluation of skeletal muscle functional parameters and body composition. Once SO has been diagnosed, a two-stage classification is applied. Stage I refers to the absence of complications attributable to altered body composition and impaired muscle function, whereas stage II is defined by the presence of at least one complication associated with altered body composition or muscle function [20]. Current evidence is insufficient to identify the optimal treatment for SO, and further research is required. Current management is primarily based on dietary therapy combined with appropriately selected physical activity. By contrast, robust evidence supporting the effectiveness of pharmacotherapy specifically for SO remains lacking, highlighting the need for further investigation and continued development in this field [20].

## 6. GLP-1 and GLP-1-Based Pharmacotherapies

GLP-1 is an incretin hormone generated through post-translational processing of proglucagon. It is secreted primarily by enteroendocrine L cells located in the distal ileum and colon, with smaller amounts produced by pancreatic α-cells and neurons within the central nervous system [3,24]. GLP-1 is released into the circulation shortly after food intake and is responsible for the incretin effect, which enhances glucose-dependent insulin secretion following oral glucose ingestion. In addition, GLP-1 suppresses glucagon secretion, delays gastric emptying, and promotes satiety, thereby reducing energy intake [24]. The biological actions of GLP-1 are mediated through activation of the glucagon-like peptide-1 receptor (GLP-1R), a member of the class B G protein-coupled receptor family. GLP-1Rs are expressed in multiple tissues, including the pancreas, gastrointestinal tract, and central nervous system. Binding of GLP-1 to the extracellular domain of GLP-1R activates G proteins and initiates intracellular signaling cascades. One of the principal signaling pathways involves activation of adenylate cyclase, resulting in increased intracellular cyclic adenosine monophosphate (cAMP) levels, which mediate many of the physiological effects of GLP-1 [24]. Despite its favorable metabolic effects, endogenous GLP-1 has a very short biological half-life of approximately 2–3 min because it is rapidly degraded by the enzyme dipeptidyl peptidase-4 (DPP-4), thereby limiting its therapeutic applicability [3]. The short half-life of native GLP-1, together with advances in peptide engineering, led to the development of GLP-1RAs. Structural modifications that increase resistance to DPP-4-mediated degradation, combined with fatty acid acylation enabling reversible albumin binding, have resulted in compounds with substantially prolonged pharmacokinetic profiles and sustained biological activity. GLP-1RAs were initially developed for the treatment of type 2 diabetes mellitus because of their potent incretin effect and glucose-lowering properties [3]. However, their therapeutic benefits extend beyond glycemic control. By delaying gastric emptying, enhancing satiety, and suppressing appetite, GLP-1RAs promote clinically meaningful weight loss. Consequently, these agents have become an important therapeutic option for obesity management in both individuals with type 2 diabetes and those without concomitant disturbances in glucose metabolism [25]. Currently, GLP-1RAs are among the most effective pharmacological therapies for obesity and are widely used in routine clinical practice [3]. The clinical success of exenatide, followed by liraglutide, paved the way for the development of newer GLP-1RAs, including semaglutide, dulaglutide, and lixisenatide. Among the currently available GLP-1RAs, semaglutide has demonstrated superior efficacy in improving glycemic control and promoting weight loss [25]. Continued advances in incretin-based therapies have also led to the development of multi-receptor agonists. Tirzepatide, a dual GIP/GLP-1RAs, represents the first agent in this class and has demonstrated remarkable efficacy in the treatment of obesity [3].

## 7. Potential Mechanisms Through Which GLP-1RAs May Modulate Sarcopenia Risk

### 7.1. Potential Molecular Mechanisms Through Which GLP-1RAs May Counteract the Development of Sarcopenia

The principal mechanisms involved in the pathophysiology of sarcopenia reinforce one another, creating a vicious cycle that contributes to disease progression. GLP-1RAs exert a broad range of biological effects and may modulate several of these processes, potentially limiting the development and progression of sarcopenia. GLP-1RAs improve insulin sensitivity in skeletal muscle cells by increasing the expression of glucose transporter type 4 (GLUT4) [26,27] and phosphorylated Akt (*p*-Akt). This stimulates the phosphoinositide 3-kinase/protein kinase B (PI3K/Akt) signalling pathway and subsequently activates the mechanistic target of rapamycin (mTOR), which regulates muscle protein synthesis. At the same time, indirect suppression of the ubiquitin–proteasome system (UPS) and the autophagy–lysosomal system may reduce muscle protein degradation. By stimulating Akt signalling, GLP-1RAs may also reduce myostatin activation by inhibiting the binding of nuclear factor kappa B (NF-κB) to the myostatin promoter. Furthermore, GLP-1RAs promote the expression of the myogenic regulatory factors MyoD and MyoG while suppressing the transcription of the ubiquitin ligases MAFbx and MuRF1 [27]. It may be hypothesized that GLP-1RAs indirectly influence the activity of insulin-like growth factor 1 (IGF-1), which regulates anabolic and catabolic pathways in skeletal muscle, through the stimulation of GLP-1 signalling [28] and growth hormone (GH) secretion [29]. IGF-1 modulates key pathways involved in muscle protein synthesis, including PI3K/Akt/mTOR and PI3K/Akt/GSK3β. Activation of PI3K/Akt signalling inhibits forkhead box O (FoxO) transcription factors, thereby limiting the transcription of E3 ubiquitin ligases that initiate UPS-mediated protein degradation. It has also been suggested that IGF-1/Akt signalling may attenuate the effects of cytokines and myostatin that promote muscle atrophy by suppressing NF-κB and Smad signalling [30]. GLP-1RAs may attenuate inflammation by reducing the production of pro-inflammatory cytokines through the activation of cyclic adenosine monophosphate–protein kinase A (cAMP–PKA) and AMP-activated protein kinase (AMPK) pathways [15,16]. Pro-inflammatory cytokines activate signalling pathways such as NF-κB, Janus kinase/signal transducer and activator of transcription (JAK/STAT), and mitogen-activated protein kinase (MAPK), disrupting the balance between muscle protein synthesis and degradation [27]. In addition, pro-inflammatory cytokines promote the expression of genes encoding E3 ubiquitin ligases, thereby stimulating UPS activity [26,27]. GLP-1RAs may also affect mitochondrial function through the AMPK–SIRT1–PGC-1α pathway [26,27]. Activation of AMPK increases the expression of peroxisome proliferator-activated receptor gamma coactivator 1-alpha (PGC-1α), a major regulator of mitochondrial biogenesis. AMPK activation also increases nicotinamide adenine dinucleotide (NAD+) availability, thereby stimulating sirtuin 1 (SIRT1), which subsequently enhances PGC-1α activity and establishes an interconnected regulatory axis. This signalling axis contributes to the efficiency of energy metabolism in skeletal muscle cells, oxidative metabolic homeostasis, and mitochondrial biogenesis [27]. Despite the available preclinical and clinical evidence, substantial uncertainty remains regarding the effects of GLP-1RAs on skeletal muscle. The proposed direct and indirect mechanisms through which these agents may influence sarcopenia risk therefore require further investigation.

### 7.2. Effects of Weight Loss on the Modulation of Sarcopenic Obesity Risk

Loss of adipose tissue induced by an appropriately designed diet or exercise intervention may improve skeletal muscle function by reducing intramuscular fat accumulation [19], increasing IGF-1 concentrations [31], and attenuating inflammation [32,33,34]. These changes may improve insulin sensitivity [34,35,36] and mitochondrial function [34]. Collectively, these multidimensional effects may reduce the risk or progression of sarcopenic obesity (SO) [19].

GLP-1RA treatment is generally associated with substantial weight loss [37]. This raises the question of whether these agents may limit the development of SO through their direct molecular effects or act primarily indirectly through adipose tissue loss and improvements in metabolic health. Current evidence does not provide a definitive answer. Randomized clinical trials directly comparing the effects of GLP-1RAs with non-pharmacological weight-loss strategies on SO-related outcomes remain lacking. Further appropriately designed studies are therefore required.

### 7.3. Potential Pathways Leading to Sarcopenia During GLP-1RA Treatment

The mechanisms through which GLP-1RAs affect skeletal muscle remain incompletely understood. Although preclinical findings suggest potentially protective effects against sarcopenia, clinical evidence does not consistently support these effects, highlighting an important knowledge gap [38]. A prolonged negative energy balance promotes not only adipose tissue loss but also the catabolism of lean tissues. Approximately 25% of the weight lost during GLP-1RA treatment may consist of FFM, although this proportion varies between studies. A sustained energy deficit may affect IGF-1 activity, potentially disrupting PI3K/Akt/mTOR signalling and muscle protein synthesis. Reduced energy intake may also impair mitochondrial biogenesis and oxidative capacity, thereby compromising muscle function. An insufficient energy supply may stimulate the hypothalamic–pituitary–adrenal axis and increase cortisol production. Elevated cortisol may counteract the anabolic effects of insulin and promote muscle proteolysis through the ubiquitin–proteasome pathway, further contributing to the loss of lean tissue. Appetite suppression induced by GLP-1RAs may also reduce protein intake, potentially increasing susceptibility to FFM loss. Furthermore, some patients experience gastrointestinal adverse effects, including vomiting, diarrhoea, or constipation, which may exacerbate the energy deficit, contribute to malnutrition, and potentially increase sarcopenia risk [37]. A prolonged negative energy balance during treatment may also contribute to fatigue and reduced physical activity, which is an important stimulus for muscle protein synthesis [29]. Individuals at increased baseline risk of sarcopenia may be particularly vulnerable to potential adverse effects during GLP-1RA therapy. These groups include older adults, postmenopausal women, individuals with low baseline muscle reserves [39], and patients with chronic conditions such as type 2 diabetes mellitus, chronic kidney disease, cardiovascular disease, heart failure, and metabolic disorders associated with liver disease [40]. The alterations in body composition and hormonal status, lower physical activity, chronic inflammation [39,40,41], and metabolic disturbances [40] characteristic of these conditions may increase susceptibility to additional challenges arising during GLP-1RA treatment.

An individualized and comprehensive approach should therefore be adopted during GLP-1RA therapy, incorporating appropriate nutritional and exercise strategies to minimize the potential risk of sarcopenia. Because of increased catabolism and anabolic resistance during pharmacological weight-loss treatment, protein intakes of 1.2–1.6 g/kg/day have been proposed, with intakes of up to 2.0 g/kg/day considered in individuals at particularly high risk of sarcopenia [39]. Protein stimulates muscle protein synthesis through activation of the mTOR complex 1 (mTORC1) pathway [42]. The 20–30% reduction in total food intake that may occur during GLP-1RA treatment could also contribute to micronutrient deficiencies. To prevent deficiencies that may adversely affect patient health, micronutrient status should be monitored and targeted supplementation introduced when a deficiency is identified [41]. Dietary patterns may also include sources of antioxidants and omega-3 fatty acids, which may help attenuate inflammation involved in the pathophysiology of sarcopenia [42]. If gastrointestinal symptoms develop, adequate dietary fibre and fluid intake should be ensured, taking the nature and severity of the symptoms into account [37]. Appropriately prescribed physical activity, adapted to the patient’s needs and functional capacity, may additionally support the maintenance of muscle mass during treatment [39]. Resistance exercise promotes IGF-1/Akt/mTORC1 signalling, suppresses autophagy and activation of the ubiquitin–proteasome pathway, and stimulates satellite cell proliferation. Endurance exercise promotes PGC-1α activity and improves insulin sensitivity, thereby supporting mitochondrial biogenesis [42].

### 7.4. Long-Term Risk of Sarcopenia

Weight regain is frequently observed following the discontinuation of GLP-1RA therapy. It has been suggested that individuals undergoing repeated cycles of weight loss and regain may experience a disproportionate recovery of FM relative to FFM, potentially contributing to the development of sarcopenia or SO [37,43]. Weight regain cannot be attributed solely to inadequate adherence to treatment recommendations. Metabolic adaptations that reduce energy expenditure and persistent changes in gut hormones regulating food intake also play important roles following weight loss [44,45]. Repeated weight cycling may additionally disrupt adipokine production and secretion and exacerbate chronic inflammation [46], potentially contributing to lower muscle mass and the development of SO [41,47,48]. Appropriate education of healthcare professionals is essential for minimizing the potential risk of SO because informed clinical management during and after treatment may help attenuate the metabolic consequences of weight regain. Evidence regarding the effects of long-term GLP-1RA treatment and treatment reinitiation after weight regain on body composition remains limited [43], highlighting the need for further research.

## 8. Individual Factors That May Influence GLP-1RA Effects and Sarcopenia Risk

Differences between women and men in body composition, hormonal profiles, and fat metabolism may substantially modify both sarcopenia risk and the response to GLP-1RA treatment [49,50,51,52]. Women generally have lower muscle mass, greater intramuscular fat deposition, and a greater capacity for the re-esterification of free fatty acids, whereas men tend to utilize free fatty acids through oxidation to a greater extent [19]. In addition, the greater muscle mass observed in men promotes the hydrolysis of triglyceride-rich lipoproteins, contributing to lower circulating triglyceride concentrations than those observed in women. These sex-related metabolic differences may influence susceptibility to sarcopenia [49].

Although ageing increases the risk of sarcopenia in both sexes, sex-specific age-related changes may modify the magnitude and timing of this risk [49,50]. During the perimenopausal period, women frequently experience an increase in FM, partly as a compensatory mechanism supporting oestrogen availability [50]. These changes may be accompanied by a more rapid decline in muscle strength and mass. At later stages of ageing, men may become increasingly susceptible to muscle loss because of declining testosterone and IGF-1 concentrations [49,50]. Some evidence also indicates a greater age-related decline in muscle mass among men than among women, reflecting sex-specific patterns of muscle ageing. Sex may also modify the response to GLP-1RA therapy. Women generally achieve greater weight loss during GLP-1RA treatment than men, which may partly result from greater drug exposure and slower drug clearance. A potential synergistic interaction between GLP-1RA activity and oestrogens in the regulation of appetite and leptin sensitivity has also been suggested. Furthermore, gastrointestinal adverse effects are reported more frequently in women, potentially contributing to a greater reduction in energy intake [51,52]. Nevertheless, the mechanisms underlying sex differences in the response to GLP-1RAs and in sarcopenia development remain incompletely understood and require further well-designed randomized studies.

Another potentially important factor is the gut microbiome. No two individuals have identical gut microbiome profiles. The composition of the gut microbiota is influenced by numerous factors, including age, ethnicity, geographical location, health status, and lifestyle [53]. Gut microorganisms exert multidimensional effects on health by modulating numerous metabolic and physiological processes [53,54,55]. Emerging evidence suggests bidirectional relationships among the gut microbiota, the activity of GLP-1-based therapies, and the risk of sarcopenia [53,55]. Through their metabolites, particularly short-chain fatty acids (SCFAs), gut microorganisms may influence the pharmacological effects of GLP-1RAs. Conversely, GLP-1RAs may directly or indirectly modulate the composition of the gut microbiota by altering energy balance, bile acid metabolism, and gastrointestinal motility. However, changes in the microbiota profile observed during treatment may also result from lifestyle modification and weight loss rather than from the direct pharmacological action of the drug. The long-term consequences of GLP-1RA-associated changes in gut microbiota composition remain incompletely understood and require further investigation [53]. Gut dysbiosis is associated with obesity, type 2 diabetes mellitus, and insulin resistance. Emerging evidence suggests that the microbiome may influence muscle function through the integrated gut–microbiota–muscle and gut–adipose tissue–muscle axes. Disruption of these interconnected pathways may contribute to the development and progression of SO. Gut dysbiosis is associated with increased inflammation and impaired intestinal barrier integrity. Increased circulating lipopolysaccharide (LPS) concentrations activate Toll-like receptor 4 (TLR4) and NF-κB signalling, leading to increased production of pro-inflammatory cytokines, insulin resistance, and enhanced muscle protein catabolism. Bile acids may modulate energy expenditure in skeletal muscle cells through activation of the Takeda G protein-coupled receptor 5 (TGR5), while activation of the farnesoid X receptor (FXR) may regulate lipid deposition in skeletal muscle. SCFAs play an important role in the gut–muscle axis by modulating glucose and lipid homeostasis and inflammatory responses. Butyrate participates in energy metabolism by entering the tricarboxylic acid cycle as acetyl-CoA and serves as the principal energy source for colonocytes. It also contributes to the regulation of inflammatory responses and maintenance of intestinal barrier integrity. Propionate and acetate may influence insulin sensitivity, partly through their involvement in hepatic glucose metabolism [55]. The interactions among the gut microbiota, GLP-1RA therapy, and sarcopenia remain incompletely understood, and their long-term consequences are uncertain. Further research is therefore needed to clarify these relationships.

Interindividual variability emphasizes the importance of personalized selection of GLP-1RA therapy and the implementation of appropriately tailored interventions to minimize potential adverse effects on skeletal muscle and sarcopenia risk.

## 9. GLP-1RAs and the Risk of Sarcopenia in Obesity: Evidence from Preclinical and Clinical Studies

Clinical studies involving individuals with overweight or obesity have demonstrated that once-weekly subcutaneous administration of semaglutide results in substantial weight loss. Dual-energy X-ray absorptiometry (DXA) analyses revealed reductions in both FM and FFM. However, despite the modest decline in FFM, its relative proportion increased owing to the greater reduction in FM, resulting in a more favorable body composition. These changes were accompanied by improvements in metabolic parameters [56,57] and physical functioning [57]. Furthermore, a phase III randomized placebo-controlled trial involving 82 women with obesity and polycystic ovary syndrome (PCOS) showed that liraglutide (3 mg/day) significantly reduced FM without producing statistically significant changes in FFM [58]. Several clinical studies involving patients with obesity and obesity-related comorbidities, including cardiovascular disease (CVD), type 2 diabetes mellitus, hypertension, dyslipidemia, and obstructive sleep apnea, have reported reductions in body weight, total FM, and visceral adipose tissue during semaglutide treatment. Although a modest reduction in skeletal muscle mass has also been reported, this decline appears to stabilize over time [59,60,61,62,63,64]. Functional outcomes were heterogeneous. Some studies reported improvements in grip strength or physical functioning [61,62], whereas others found no significant changes in grip strength, chair-rise performance, gait speed, or muscle quality [59,63,64]. Moreover, one study in older adults reported lower handgrip strength and gait speed after 24 months of treatment [65]. Moreover, the proportion of FFM relative to total body weight increased, suggesting that the overall weight loss was primarily driven by reductions in adipose tissue rather than lean tissue [59,60,61,62,63,64].

Prospective studies conducted by Alissou et al. [62] and Volpe et al. [64] yielded mixed findings regarding sarcopenia-related outcomes during semaglutide treatment. In a prospective study involving 48 patients with obesity and type 2 diabetes mellitus, semaglutide treatment resulted in reductions in body weight, total FM, visceral adipose tissue, and skeletal muscle mass. A decrease in the skeletal muscle index (SMI) exceeding 40% was observed in 18% of participants. Importantly, neither grip strength nor the muscle quality index changed significantly between baseline and the end of treatment. Furthermore, although the skeletal muscle mass-to-bioimpedance-assessed visceral adipose tissue ratio (SMM/BIA-VAT) increased, this finding should not be interpreted as evidence that muscle health was preserved or that sarcopenia risk was absent [64]. The SEMALEAN study further demonstrated that 12 months of semaglutide therapy reduced the prevalence of sarcopenic obesity by 16%. Among participants with sarcopenic obesity at baseline, 22% no longer met the diagnostic criteria following treatment. In addition, 49% of participants maintained a non-sarcopenic phenotype throughout the follow-up period, whereas only 5% developed sarcopenic obesity during therapy [62]. These findings are encouraging but should be interpreted cautiously, as the relatively small number of participants who developed sarcopenic obesity does not exclude a clinically meaningful risk in individuals with greater baseline vulnerability. In particular, older adults, frail individuals, and patients with inadequate nutritional intake may be at greater risk, but such populations remain insufficiently represented in the available evidence [14,66]. A randomized clinical trial involving individuals with overweight or obesity and metabolic syndrome (MetS) demonstrated that liraglutide improved skeletal muscle composition independently of weight loss. After 40 weeks of treatment, magnetic resonance imaging (MRI) revealed a 2.87% reduction in thigh muscle fat infiltration accompanied by only a modest decrease in muscle volume. Furthermore, the proportion of participants presenting with adverse muscle composition decreased by 2.8% [67]. Similarly, the SLIM LIVER study demonstrated a 3.6% reduction in psoas muscle fat content following semaglutide treatment among participants aged 40–60 years. In contrast, individuals younger than 40 years exhibited an increase in psoas muscle fat, which the authors suggested might be related to a greater absolute reduction in fasting triglyceride concentrations [63]. The main findings of clinical studies evaluating the effects of GLP-1RAs on skeletal muscle and sarcopenia-related outcomes in individuals with overweight or obesity are summarized in Table 1.

## 10. Discussion

### 10.1. Clinical Interpretation and Biological Mechanisms of GLP-1RA-Induced Changes in Skeletal Muscle

The available clinical evidence demonstrates that treatment with GLP-1RAs results in substantial weight loss, primarily attributable to a reduction in adipose tissue. Across studies evaluating semaglutide and liraglutide, pronounced reductions in FM were reported, whereas reductions in FFM were generally smaller [56,57,58]. These findings suggest that, at the group level, reductions in FFM were smaller than reductions in adipose tissue. Moreover, several studies reported an increase in the relative proportion of FFM despite an absolute decrease in lean tissue, reflecting a more favorable body composition following treatment [56,57]. Nevertheless, the interpretation of FFM changes requires caution when assessing the risk of sarcopenia. FFM comprises not only skeletal muscle but also internal organs, bone tissue, body fluids, and water contained within adipose tissue [17]. Consequently, reductions in FFM cannot be directly equated with skeletal muscle loss, although they do not rule it out either. It is also worth noting that studies do not assess the direct impact of treatment on skeletal muscle mass [56,57]. Weight loss is associated with a reduction in muscle mass; however, the extent to which it may influence the development or progression of sarcopenia depends on the patient’s muscle reserves [69]. Another important consideration is the methodology used for body composition assessment. In the majority of clinical studies, FM and FFM were measured using DXA. Although DXA is widely available and frequently used in clinical research, measurements may vary between different manufacturers and scanner models, potentially limiting the comparability of results. In contrast, magnetic resonance imaging (MRI) and computed tomography (CT) are currently regarded as the gold standard for the assessment of skeletal muscle quantity and morphology [14]. These imaging techniques also enable accurate evaluation of intramuscular fat infiltration, an important feature associated with impaired muscle quality and sarcopenia [17]. However, their routine application remains limited because of high equipment costs, limited availability, and the requirement for specialized personnel [14]. Consequently, the lack of advanced imaging techniques in most published studies makes it difficult to draw definitive conclusions regarding the direct effects of GLP-1RAs on the development of sarcopenia.

Despite the modest reductions in FFM reported in several clinical studies, the available evidence does not demonstrate a consistent deterioration in muscle strength or physical performance. However, these findings should be interpreted cautiously. In particular, the absence of statistically significant changes in measures such as grip strength or muscle quality should not be interpreted as evidence that skeletal muscle health is preserved. Null findings may reflect limited statistical power, relatively short follow-up, measurement variability or insensitivity, and the characteristics of the study populations. Most available studies were not specifically designed or powered to detect changes in sarcopenia-related outcomes, and functional assessments were often secondary rather than primary endpoints. Therefore, the absence of a statistically significant decline in muscle function does not exclude a clinically meaningful deterioration in susceptible individuals [59,60,61,62,63,64]. Moreover, preserved short-term muscle function does not establish that a reduction in muscle reserve is clinically irrelevant. Reduced muscle reserve may increase vulnerability during ageing, acute illness, hospitalization, falls, or treatment discontinuation, particularly in older, frail, or nutritionally vulnerable individuals. Preclinical studies provide evidence for potentially beneficial effects of GLP-1RAs on skeletal muscle through multiple mechanisms, including the stimulation of myogenic pathways and attenuation of chronic inflammation [70]. However, these mechanistic findings cannot substitute for clinical evidence regarding sarcopenia risk. Therefore, the potential biological effects of GLP-1RAs on skeletal muscle should be considered hypothesis-generating rather than sufficient evidence of preserved muscle health in patients receiving these therapies. In selected prospective studies evaluating semaglutide, a reduction in the prevalence and severity of sarcopenic obesity was observed, with some patients no longer fulfilling the diagnostic criteria following treatment [62,64]. However, these findings should be interpreted cautiously and should not be considered evidence that GLP-1RA therapy eliminates or substantially reduces the risk of sarcopenia in the broader population. The relatively small number of participants who developed sarcopenic obesity during follow-up does not exclude a clinically meaningful risk in individuals with greater baseline vulnerability. In particular, older adults, frail individuals, and patients with inadequate nutritional intake may be at greater risk, but such populations remain insufficiently represented in the available evidence [14]. Thus, the current studies do not allow the risk of sarcopenia to be reliably excluded, particularly in clinically vulnerable subgroups. Taken together, the currently available evidence suggests that the evaluation of sarcopenia risk during GLP-1RA therapy should not rely exclusively on changes in muscle mass. Instead, a comprehensive assessment should also include muscle strength, physical performance, muscle quality, intramuscular fat infiltration and other clinically relevant measures of skeletal muscle health [63,64,67,71,72]. However, the limited and heterogeneous nature of the available evidence means that the absence of adverse changes in individual measures should not be interpreted as evidence that sarcopenia risk has been excluded.

Although the precise mechanisms responsible for the effects of GLP-1RAs on skeletal muscle have not yet been fully elucidated, preclinical studies suggest the involvement of several complementary biological pathways. Among these, activation of the sirtuin 1 (SIRT1) signaling pathway appears to be particularly important. SIRT1 plays a pivotal role in maintaining skeletal muscle homeostasis by regulating mitochondrial function, energy metabolism, oxidative stress, and muscle protein turnover. In experimental models of obesity, adipose tissue hypertrophy and metabolic dysfunction have been associated with reduced SIRT1 activity, increased expression of the muscle atrophy marker Atrogin-1, and decreased myogenin expression. Xiang et al. demonstrated that liraglutide and semaglutide enhanced SIRT1 signaling, increased myogenin and GLUT4 expression, and suppressed Atrogin-1 expression, thereby attenuating skeletal muscle atrophy [71]. Complementary studies showed that liraglutide and exendin-4 reduced the expression of MuRF1, MAFbx/Atrogin-1, and myostatin while promoting Akt-dependent myogenic differentiation [73,74]. However, more recent findings indicate that semaglutide may also increase the expression of proteolysis-related genes, including *Fbxo32*, Atrogin-1, and *Klf15*, under conditions of marked weight loss [75]. These findings suggest that the effects of GLP-1RAs on muscle protein turnover may depend on the experimental model, metabolic context, and magnitude of the energy deficit. Another potential mechanism involves the regulation of muscle protein turnover through the inhibition of proteolysis and the stimulation of myogenesis. Liraglutide, semaglutide, and exendin-4 have all been shown to reduce the expression of Atrogin-1 and MuRF-1, two key E3 ubiquitin ligases responsible for muscle protein degradation, while simultaneously increasing the expression of the myogenic regulatory factors myogenin and MyoD, thereby promoting muscle regeneration and differentiation [71,74]. However, not all preclinical findings support an unequivocally protective effect of GLP-1RAs on skeletal muscle. Jeromson et al. reported that semaglutide induced reductions in muscle fiber size and muscle strength comparable to those observed during caloric restriction, despite producing greater reductions in adipose tissue. In addition, semaglutide increased the expression of several genes involved in proteolysis, including *Fbxo32*, Atrogin-1, and *Klf15*, suggesting activation of catabolic pathways within skeletal muscle [75]. These observations indicate that the effects of semaglutide on skeletal muscle may depend on the metabolic context, severity of the energy deficit, and experimental conditions, and therefore may not always exert a uniformly protective effect. Overall, the available evidence suggests that the interaction between GLP-1RA therapy and skeletal muscle metabolism is complex and likely influenced by multiple physiological and pathological factors, warranting further investigation in both experimental and clinical settings.

### 10.2. Mitochondrial Function and Intramuscular Fat Accumulation

Mitochondrial dysfunction is considered one of the key mechanisms underlying the pathophysiology of sarcopenia [15]. Accordingly, several preclinical studies have investigated the effects of GLP-1RAs on mitochondrial function in skeletal muscle [76,77,78]. However, the available evidence remains inconsistent. Choi et al. demonstrated that semaglutide administration in mice with obesity improved skeletal muscle oxidative phosphorylation (OXPHOS) efficiency despite the absence of significant alterations in the expression of most mitochondrial complex proteins [77]. In contrast, Choung et al. reported that exendin-4 increased oxygen consumption and upregulated genes involved in fatty acid oxidation and thermogenesis, including UCP1 and PPARα, suggesting enhanced mitochondrial energy metabolism in skeletal muscle cells [76]. Old et al. investigated the effects of semaglutide, tirzepatide, and cagrilintide on mitochondrial function in C2C12 myotubes. These compounds differ in their pharmacological mechanisms: semaglutide is a GLP-1 analogue acting as a selective GLP-1RA [79], tirzepatide is an imbalanced dual GIP and GLP-1RAs with greater engagement of the GIP receptor than the GLP-1R [80], whereas cagrilintide is a long-acting dual amylin and calcitonin receptor agonist [81]. Although cagrilintide falls outside the scope of pharmacotherapy covered in this review, the study included this compound alongside semaglutide and tirzepatide. Semaglutide initially induced a transient reduction in mitochondrial respiration and ATP production. In contrast, although tirzepatide also caused an early suppression of mitochondrial activity, prolonged exposure for five days resulted in improved mitochondrial respiration, ATP production, maximal respiratory capacity, and oxidative coupling efficiency [78]. The discrepancies among these findings may largely reflect differences in experimental models. Whereas Choi et al. employed an in vivo model of obesity, Old et al. performed experiments using cultured skeletal muscle cells. Cell culture models do not fully reproduce the complex endocrine and metabolic interactions occurring in vivo, which may partly explain the divergent observations. Collectively, these findings suggest that the effects of GLP-1RAs on mitochondrial function are likely influenced by the specific pharmacological agent, duration of treatment, and experimental model employed. Beyond their effects on mitochondrial biology, GLP-1RAs have been associated with improvements in metabolic health. Both preclinical and clinical studies have demonstrated enhanced insulin sensitivity together with reductions in triglyceride concentrations, LDL cholesterol levels, and systemic inflammatory markers following GLP-1RA therapy [58,59,63,71,72]. Given the well-established links between chronic inflammation, insulin resistance, mitochondrial dysfunction, and skeletal muscle deterioration, these metabolic improvements may indirectly contribute to the preservation of muscle function and quality. Intramuscular fat infiltration represents another important feature of obesity-related sarcopenia. Excess lipid accumulation within skeletal muscle disrupts muscle architecture, cellular metabolism, and intracellular signaling pathways, ultimately impairing muscle function and physical performance [18]. This phenomenon is frequently observed in individuals with obesity [66] and is considered a hallmark of sarcopenic obesity [18]. The available evidence suggests that GLP-1RAs may beneficially influence muscle quality by reducing intramuscular fat accumulation. In the randomized clinical trial conducted by Pandey et al., 40 weeks of liraglutide treatment significantly reduced thigh muscle fat content as assessed by magnetic resonance imaging. Importantly, this improvement occurred despite only a modest reduction in muscle volume, indicating that muscle quality can improve independently of changes in muscle mass [66]. Similar findings were reported in the SLIM LIVER study, in which semaglutide treatment reduced psoas muscle fat content in participants aged 40–60 years [62]. Interestingly, this effect was not observed in younger participants, suggesting that age and baseline metabolic status may influence the skeletal muscle response to GLP-1RA therapy. These clinical observations are supported by preclinical evidence. Xiang et al. demonstrated that liraglutide and semaglutide reduced intramyocellular lipid droplet accumulation in skeletal muscle of obese mice [71]. Likewise, exendin-4 attenuated lipid accumulation in muscle cells by activating pathways involved in cellular energy metabolism [76]. These findings suggest that intramuscular fat may decrease despite modest reductions in muscle volume; however, current evidence does not establish that these changes preserve muscle function or reduce sarcopenia risk.

### 10.3. Limitations and Future Directions

Several limitations of the currently available evidence should be acknowledged. First, most clinical studies assessed body composition using DXA, whereas only a limited number employed magnetic resonance imaging, which is currently considered the gold standard for the assessment of skeletal muscle quantity, morphology, and intramuscular fat infiltration [14,62,66]. Consequently, the available evidence may not fully capture qualitative changes in skeletal muscle occurring during GLP-1RA therapy. Another important limitation is the considerable heterogeneity of the included studies. The investigated populations differed substantially with respect to age, obesity severity, comorbidities, and concomitant pharmacological treatment. Furthermore, studies evaluated different GLP-1RAs administered at varying doses and treatment durations, making direct comparisons across studies challenging [55,56,57,58,59,60,61,62,63,64,65,66]. It should also be emphasized that most available studies primarily focused on changes in body weight and body composition, whereas relatively few assessed clinically relevant functional outcomes such as muscle strength, physical performance, or exercise capacity. Importantly, the current evidence base is not sufficiently designed to exclude an increased risk of sarcopenia during GLP-1RA therapy. Functional and muscle-related outcomes, including grip strength, chair-rise performance, gait speed, and muscle quality, were assessed inconsistently across studies and were often secondary rather than primary outcomes. Therefore, statistically non-significant changes in these measures should not be interpreted as evidence of preserved muscle health, as studies may have been limited by sample size, follow-up duration, measurement characteristics, and selected study populations [14,64]. As highlighted by Neeland et al. [17], future research should incorporate comprehensive assessments of skeletal muscle function in addition to conventional body composition measurements. Well-designed prospective clinical studies using standardized imaging techniques, validated measures of muscle quality and function, and long-term follow-up are therefore needed to clarify the effects of GLP-1RAson sarcopenia risk and skeletal muscle health.

## 11. Conclusions

GLP-1RAs induce substantial weight loss that is primarily driven by reductions in adipose tissue, whereas decreases in FFM are considerably less pronounced. A reduction in FFM observed during GLP-1RA therapy alone is insufficient to establish the development of sarcopenia, which requires a comprehensive assessment of muscle mass, strength, and physical performance. Although several clinical studies have reported preserved or improved muscle-related functional outcomes despite modest reductions in lean mass, the absence of statistically significant deterioration in these measures should not be interpreted as evidence that muscle health is preserved. Preclinical evidence suggests that GLP-1RAs may exert beneficial effects on skeletal muscle through multiple mechanisms, including activation of the SIRT1 signaling pathway, inhibition of proteolysis, preservation of mitochondrial function, and anti-inflammatory and metabolic effects. However, these findings cannot substitute for clinical evidence regarding sarcopenia risk. Most clinical studies have relied on DXA to assess body composition, a technique that does not allow precise evaluation of skeletal muscle quantity, quality, or intramuscular fat infiltration. Consequently, the current evidence is insufficient to establish whether GLP-1RAs increase, decrease, or have a neutral effect on the risk of sarcopenia. Although available studies do not consistently demonstrate deterioration in muscle strength or physical performance, the absence of statistically significant changes should not be interpreted as evidence that muscle health is preserved. The limited number of studies directly assessing sarcopenia, heterogeneous assessment methods, relatively short follow-up, and selected study populations preclude definitive conclusions and do not allow a clinically meaningful risk of sarcopenia to be excluded, particularly in older, frail, or nutritionally vulnerable individuals. Further well-designed prospective studies using standardized and comprehensive measures of skeletal muscle are needed to clarify the long-term effects of GLP-1RAs on muscle health and their potential role in preventing or modifying the progression of sarcopenia in individuals with obesity.

## Figures and Tables

**Table 1:** Clinical studies evaluating the effects of GLP-1RAs on skeletal muscle and sarcopenia-related outcomes in overweight or obesity.

Study	Study Design	Study Population	Intervention	Main Findings
McCrimmon et al., 2020 [56]	Substudy of the SUSTAIN 8 randomized controlled clinical trial	Adults with overweight/obesity and type 2 diabetes; *n* = 178	Semaglutide (1.0 mg/week); 52 weeks	Both treatments ↓ body weight, FM and FFM; semaglutide produced greater VAT reduction and improved FFM/FM ratio.
Wilding et al., 2021 [57]	Randomized, double-blind, placebo-controlled clinical trial	Adults with overweight/obesity and ≥1 obesity-related comorbidity; *n* = 1961	Semaglutide (2.4 mg/week); lifestyle intervention; 68 weeks	↓ Body weight, BMI, waist circumference, FM and VAT; modest ↓ FFM with improved FFM/FM ratio; improved physical functioning and metabolic profile; mild-to-moderate gastrointestinal symptoms not requiring treatment discontinuation (74.2%); serious adverse events (9.8%).
Elkind-Hirsch et al., 2022 [58]	Randomized, placebo-controlled phase 3 clinical trial	Women with obesity and PCOS; *n* = 82	Liraglutide (3 mg/day); 32 weeks	↓ Body weight, BMI, waist circumference and FM; no significant changes in FFM; improved HOMA-IR and lipid profile; moderate gastrointestinal symptoms (58.2%)
Xiang et al., 2023 [59]	Retrospective observational study	Adults with obesity without diabetes; *n* = 53	Semaglutide (up to 1.0 mg/week); 6 months	↓ Body weight, FM and VAT; modest ↓ muscle mass without significant changes in SMI, calf circumference or grip strength; improved glucose metabolism and HOMA-IR; mild gastrointestinal adverse events were reported.
Ozeki et al., 2022 [60]	Retrospective observational study	Patients with obesity, type 2 diabetes and cardiovascular disease; *n* = 48	Semaglutide; 3 months	↓ Body weight, BMI, FM and skeletal muscle mass; improved glycemic control.
Borlaug et al., 2023 [61]	International, randomized, double-blind, placebo-controlled clinical trial	Patients with obesity and HFpEF; *n* = 529	Semaglutide (2.4 mg/week); 52 weeks	↓ Body weight and CRP; improved symptoms and physical function (KCCQ-CSS); serious adverse events were reported in 71 patients.
Alissou et al., 2026 [62]	Prospective longitudinal study (SEMALEAN)	Adults with obesity and obesity-related comorbidities (SEMALEAN); *n* = 115	Semaglutide (up to 2.4 mg/week); 12 months	↓ Body weight, FM and VAT; transient ↓ muscle mass followed by stabilization; ↑ grip strength; ↓ prevalence of sarcopenic obesity.
Ditzenberger et al., 2025 [63]	Pilot, single-arm, open-label phase 2b clinical trial	Adults with abdominal obesity, HIV and insulin resistance/prediabetes (SLIM LIVER); *n* = 51	Semaglutide (up to 1.0 mg/week); 24 weeks	↓ Body weight and BMI; modest ↓ psoas muscle volume without significant changes in intramuscular fat; non-significant improvements in chair-rise performance and gait speed.
Volpe et al., 2022 [64]	Prospective, single-arm, real-world observational study	Adults with obesity and type 2 diabetes; *n* = 48	Semaglutide (up to 1.0 mg/week); 52 weeks	↓ Body weight, FM, FMI, VAT, FFMI and SMI; no significant changes in grip strength or muscle quality index; ↑ SMM/BIA-VAT ratio; the clinical significance of this change for muscle preservation remains uncertain.
Ren et al., 2025 [65]	Retrospective matched-cohort study	Older adults (≥65 years) with T2DM; semaglutide *n* = 220, matched controls *n* = 212	Semaglutide; 24 months	↓ BMI and ASMI, with greater muscle loss than controls. Handgrip strength and gait speed were significantly lower at 24 months; grip strength initially improved in men but subsequently declined, while gait speed decreased in both sexes. Higher semaglutide dose was associated with greater muscle loss.
Pandey et al., 2024 [67]	Secondary analysis of a randomized, double-blind, placebo-controlled clinical trial	Adults with overweight/obesity, MetS and high cardiovascular risk; *n* = 128	Liraglutide (3 mg/day); 40 weeks	↓ Body weight, FM and intramuscular fat; modest ↓ muscle volume without significant changes in muscle volume z-score; improved muscle composition independent of weight loss.
Perna et al., 2016 [68]	Prospective, single-arm pilot study	Overweight/obese older adults with T2DM; *n* = 9	Liraglutide (up to 3.0 mg/day); 24 weeks	↓ BMI, body weight, FM and android fat; SMI increased slightly (+0.03 kg/m^2^), suggesting preservation of muscle mass. No direct assessment of muscle strength or physical performance.

N, Number of subjects; BMI, body mass index; BIA-VAT, bioimpedance-assessed visceral adipose tissue; CRP, *C*-reactive protein; FFM, fat-free mass; FFMI, fat-free mass index; FM, fat mass; FMI, fat mass index; HFpEF, heart failure with preserved ejection fraction; HOMA-IR, homeostatic model assessment of insulin resistance; KCCQ-CSS, Kansas City Cardiomyopathy Questionnaire Clinical Summary Score; MetS, metabolic syndrome; PCOS, polycystic ovary syndrome; SMI, skeletal muscle index; SMM/BIA-VAT, skeletal muscle mass to bioimpedance-assessed visceral adipose tissue ratio; VAT, visceral adipose tissue; ↑ increase; ↓ decrease.

## Data Availability

No new data were created or analyzed in this study. Data sharing is not applicable to this article.

## Abbreviations

The following abbreviations are used in this manuscript:

Akt	protein kinase B
AMPK	AMP-activated protein kinase
ATP	adenosine triphosphate
BIA-VAT	bioimpedance-assessed visceral adipose tissue
BMI	body mass index
cAMP	cyclic adenosine monophosphate
CRP	C-reactive protein
CSA	cross-sectional area
CT	computed tomography
CVD	cardiovascular disease
DPP-4	dipeptidyl peptidase-4
DXA	dual-energy X-ray absorptiometry
EASO	European Association for the Study of Obesity
ESPEN	European Society for Clinical Nutrition and Metabolism
EWGSOP	European Working Group on Sarcopenia in Older People
Fbxo32	F-box protein 32 (Atrogin-1)
FKBP52	FK506-binding protein 52
FFM	fat-free mass
FFMI	fat-free mass index
FM	fat mass
FMI	fat mass index
FoxOs	forkhead box O transcription factors
FXR	farnesoid X receptor
GH	growth hormone
GIF-1	insulin-like growth factor 1
GIP	glucose-dependent insulinotropic polypeptide
GLP-1	glucagon-like peptide-1
GLP-1RA	glucagon-like peptide-1 receptor agonist
GLP-1R	glucagon-like peptide-1 receptor
GLUT4	glucose transporter type 4
HFD	high-fat diet
HFpEF	heart failure with preserved ejection fraction
HOMA-IR	homeostatic model assessment of insulin resistance
HSL	hormone-sensitive lipase
HSP70	heat shock protein 70
HSP90	heat shock protein 90
IL-1β	interleukin-1 beta
IL-6	interleukin-6
JAK/STAT	Janus kinase/signal transducer and activator of transcription
KEGG	Kyoto Encyclopedia of Genes and Genomes
KCCQ-CSS	Kansas City Cardiomyopathy Questionnaire Clinical Summary Score
Klf15	Krüppel-like factor 15
LDL-C	low-density lipoprotein cholesterol
LPS	lipopolysaccharide
LYRM7	LYR motif-containing protein 7
MAPK	mitogen-activated protein kinase
MAFbx	muscle atrophy F-box (Atrogin-1)
MASLD	metabolic dysfunction-associated steatotic liver disease
MetS	metabolic syndrome
MRI	magnetic resonance imaging
MSTN	myostatin
mtDNA	mitochondrial DNA
mTOR	mechanistic target of rapamycin
mTORC1	mechanistic target of rapamycin complex 1
MuRF1	muscle RING finger protein-1
MyoD	myogenic differentiation factor
MyoG	myogenin
NAD+	nicotinamide adenine dinucleotide
NF-κB	nuclear factor kappa-light-chain-enhancer of activated B cells
OXPHOS	oxidative phosphorylation
P/O	phosphate-to-oxygen ratio
PCOS	polycystic ovary syndrome
PGC-1α	peroxisome proliferator-activated receptor gamma coactivator 1-alpha
PI3K/Akt	phosphoinositide 3-kinase/protein kinase B Akt
PKA	protein kinase A
PPARα	peroxisome proliferator-activated receptor alpha
p23	p23 co-chaperone
SCFA	short-chain fatty acids
SIRT1	sirtuin 1
SMI	skeletal muscle index
SMM/BIA-VAT	skeletal muscle mass to bioimpedance-assessed visceral adipose tissue ratio
SO	sarcopenic obesity
TG	triglycerides
TLR4	toll-like receptor
TNF-α	tumor necrosis factor alpha
TTC19	tetratricopeptide repeat domain-containing protein 19
ROS	reactive oxygen species
UCP1	uncoupling protein-1
UPS	ubiquitin–proteasome system
VAT	visceral adipose tissue
WHO	World Health Organization
